# TanshinoneIIA and Cryptotanshinone Protect against Hypoxia-Induced Mitochondrial Apoptosis in H9c2 Cells

**DOI:** 10.1371/journal.pone.0051720

**Published:** 2013-01-14

**Authors:** Hyou-Ju Jin, Xiao-Liang Xie, Ji-Ming Ye, Chun-Guang Li

**Affiliations:** 1 Traditional & Complementary Medicine Program, RMIT Health Innovations Research Institute, School of Health Sciences, RMIT University, Bundoora, Victoria, Australia; 2 Medicinal Herb Research Center, Hebei Academy of Agricultural and Forestry Sciences, Shijiazhuang, China; 3 Center for Complementary Medicine Research, National Institute of Complementary Medicine, University of Western Sydney, Campbelltown Campus, Penrith, New South Wales, Australia; UAE University, United Arab Emirates

## Abstract

Mitochondrial apoptosis pathway is an important target of cardioprotective signalling. Tanshinones, a group of major bioactive compounds isolated from *Salvia miltiorrhiza,* have been reported with actions against inflammation, oxidative stress, and myocardial ischemia reperfusion injury. However, the actions of these compounds on the chronic hypoxia-related mitochondrial apoptosis pathway have not been investigated. In this study, we examined the effects and molecular mechanisms of two major tanshonones, tanshinone IIA (TIIA) and cryptotanshinone (CT) on hypoxia induced apoptosis in H9c2 cells. Cultured H9c2 cells were treated with TIIA and CT (0.3 and 3 μΜ) 2 hr before and during an 8 hr hypoxic period. Chronic hypoxia caused a significant increase in hypoxia inducible factor 1α expression and the cell late apoptosis rate, which was accompanied with an increase in caspase 3 activity, cytochrome c release, mitochondria membrane potential and expression of pro-apoptosis proteins (Bax and Bak). TIIA and CT (0.3 and 3 μΜ), in concentrations without affecting the cell viability, significantly inhibited the late apoptosis and the changes of caspase 3 activity, cytochrome c release, and mitochondria membrane potential induced by chronic hypoxia. These compounds also suppressed the overexpression of Bax and reduced the ratio of Bax/Bcl-2. The results indicate that TIIA and CT protect against chronic hypoxia induced cell apoptosis by regulating the mitochondrial apoptosis signaling pathway, involving inhibitions of mitochondria hyperpolarization, cytochrome c release and caspase 3 activity, and balancing anti- and pro-apoptotic proteins in Bcl-2 family proteins.

## Introduction

Myocardial hypoxia is closely associated with cardiac dysfunctions, in particular myocardial infarction (MI), ischemia reperfusion injury, and hypertrophy [1]. Hypoxia can cause changes of important cell signalling mechanisms involved in regulation of mitochondrial function, intracellular calcium and pH homeostasis, resulting in oxidative stress which subsequently induce cell death via apoptosis or/and necrosis [2].

The mitochondria dependent intrinsic apoptosis pathway plays an important role in cardiac cell injury under various pathological conditions [3]. For example, there is strong evidence that mitochondria transition pore opening (MPTP) is involved in stress induced cardiac cell damage and cell death [4]. The event of MPTP opening is affected by various factors including intracellular Ca^2+^, oxidative radicals, ATP levels and Bcl-2 family proteins [5]. MPTP opening causes a transient hyperpolarization, followed by depolarization, of the mitochondria membrane, which in turn causes the release of apoptotic proteins such as cytochrome c, smac and apoptosis inducing factor (AIF), resulting in caspase 3 activation and apoptosis. Other drivers of MPTP opening include Bcl-2 family proteins, in particular pro-apoptotic (Bax, Bak and Bad) proteins but also involving anti-apoptotic (Bcl-2, Bcl-xl) proteins [6], [7]. Cardiac cells under prolonged hypoxia condition have been shown with mitochondrial damage and apoptosis, including changes in cytochrome c release, caspase 3 activation and Bcl-2 family proteins [8]. It has been known that Bcl-2 family proteins are regulated by hypoxia inducible factor 1α (HIF-1α), a protein highly activated in low oxygen condition [9]. A previous study has reported that increased expression of HIF-1α and its regulated genes by hypoxia was associated with cell apoptosis and Bax and Bcl-xl proteins via stimulation of Bnip3 and p-53 proteins [10].

Tanshinones are a group of bioactive compounds isolated from *Salvia miltiorrhiza* (*Danshen*), a traditionally medicinal plant used for treating angina pectoris, atherosclerosis and MI [11]. Tanshinone IIA (TIIA) and cryptotanshinone (CT) are two major bioactive tanshinone [12]. They have reported with actions against inflammation, oxidative stress, myocardial infarction, and myocardial ischemia reperfusion injury [13] **.** For example, studies in neonatal cardiomyocytes have revealed a vasodilation action and anti-apoptosis effects of TIIA [14], [15] by attenuating ROS level and enhancing Bcl-2 protein expression. TIIA has also been shown with anti-inflammatory effects, by reducing monocyte chemoattractant protein-1 (MCP-1) expression in cardiac fibroblasts [16]. *In vivo* studies have demonstrated that TIIA reduced MI size, an effect was associated with inhibition of expression of tumor necrosis factor-α (TNF-α), MCP-1, and nuclear transcription factor-kappa B (NF-kappa B) [16]. Similarly, CT has been shown with anti-inflammatory actions by inhibition of expressions of NF-kB and matrix metalloproteinases-9 (MMP-9) and vasodilator actions by reduction of calcium influx or attenuating expressions of I/R injuries via suppressing TNF-α and interleukin1β in cultured cells or *in vivo* [17], [18], [19]. However, the actions of TIIA and CT on chronic hypoxia induced cardiac cell injury have still not been investigated, especially on the mechanisms involving MPTP and mitochondrial apoptosis pathway. Thus, in this study, we examined the effects and mechanisms of TIIA and CT on hypoxia induced apoptosis in H9c2 cells.

## Results

### Effects of TIIA and CT on Cell Viability in H9c2 Cells

**Figure 1:**
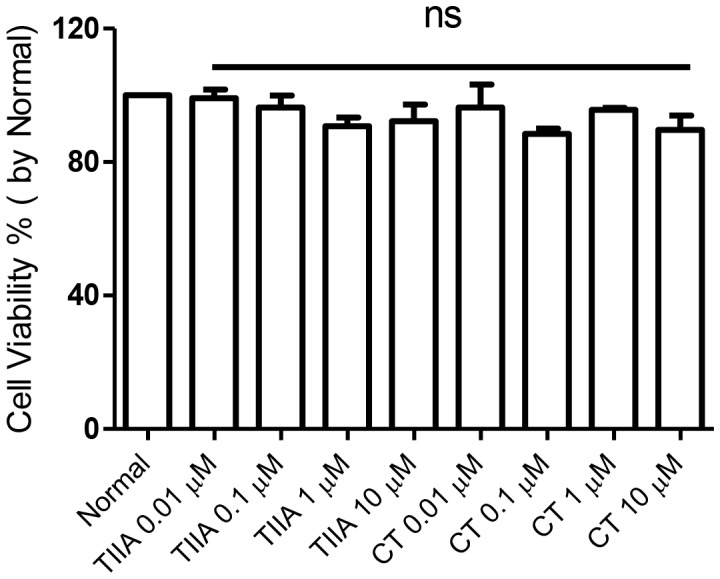
Effects of TIIA and CT on cell viability in H9c2 cells. TIIA and CT (0.01 µM -10 µM) were added and incubated for 24 hr. Cell viability was determined by MTT assay. Values are mean ± SEM from n = 3, ns: not significant compared to normal (P>0.05).

TIIA and CT (0.01–10 μΜ) did not significantly affect cell viability in H9c2 cells (P>0.05) (Figure 1).

### Effects of TIIA and CT on Hypoxia Induced HIF-1α Stabilization

**Figure 2:**
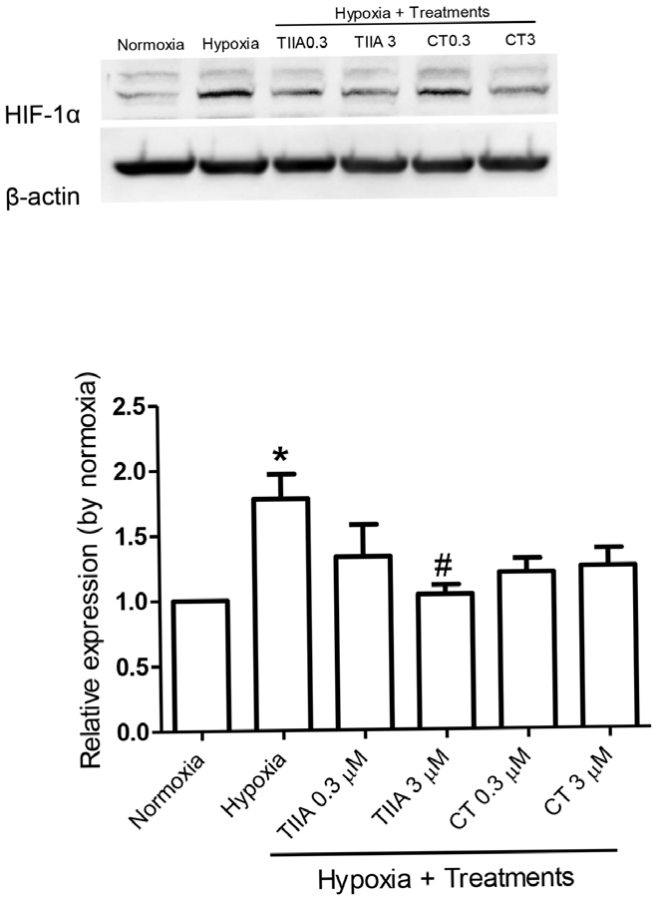
Effects of TIIA and CT on hypoxia induced HIF-1α stabilization in H9c2 cells. Equal amounts (25 µg) of the lysates were used for western blotting for HIF-1α antibody on 8% SDS-PAGE gel. Values shown are mean ± SEM from n = 4, * P<0.05 vs. normoxia, # P<0.05 vs. hypoxia.

Prolonged hypoxia can cause a distinctive change in cellular signalling by stabilization of HIF-1α. Activated HIF-1α can regulate mitochondrial proteins and interact with other signalling molecules, such as NO, to cause cell damages [2], [9]. Thus, western blot was carried out first to determine whether HIF-1α was involved in our experimental hypoxia condition, and then to determine the effects of TIIA and CT on the stabilization of HIF-1α. To specify HIF-1α translocation and stabilization, cell nuclear fractions were isolated and subjected to western blot. In the hypoxia control group, a significant increase in HIF-1α stabilization was detected with a 1.7 fold increase than that of the normoxia group. Cells treated with TIIA and CT showed a tendency of decreased expression of HIF-1α, compared to the hypoxia control, although they did not reach a statistical significance level, except TIIA 3 µM showed a significant degradation on hypoxia induced HIF-1α accumulation (from 1.7 to 1.1 fold, Figure 2).

### Effects of TIIA and CT on Hypoxia Induced Apoptosis

**Figure 3:**
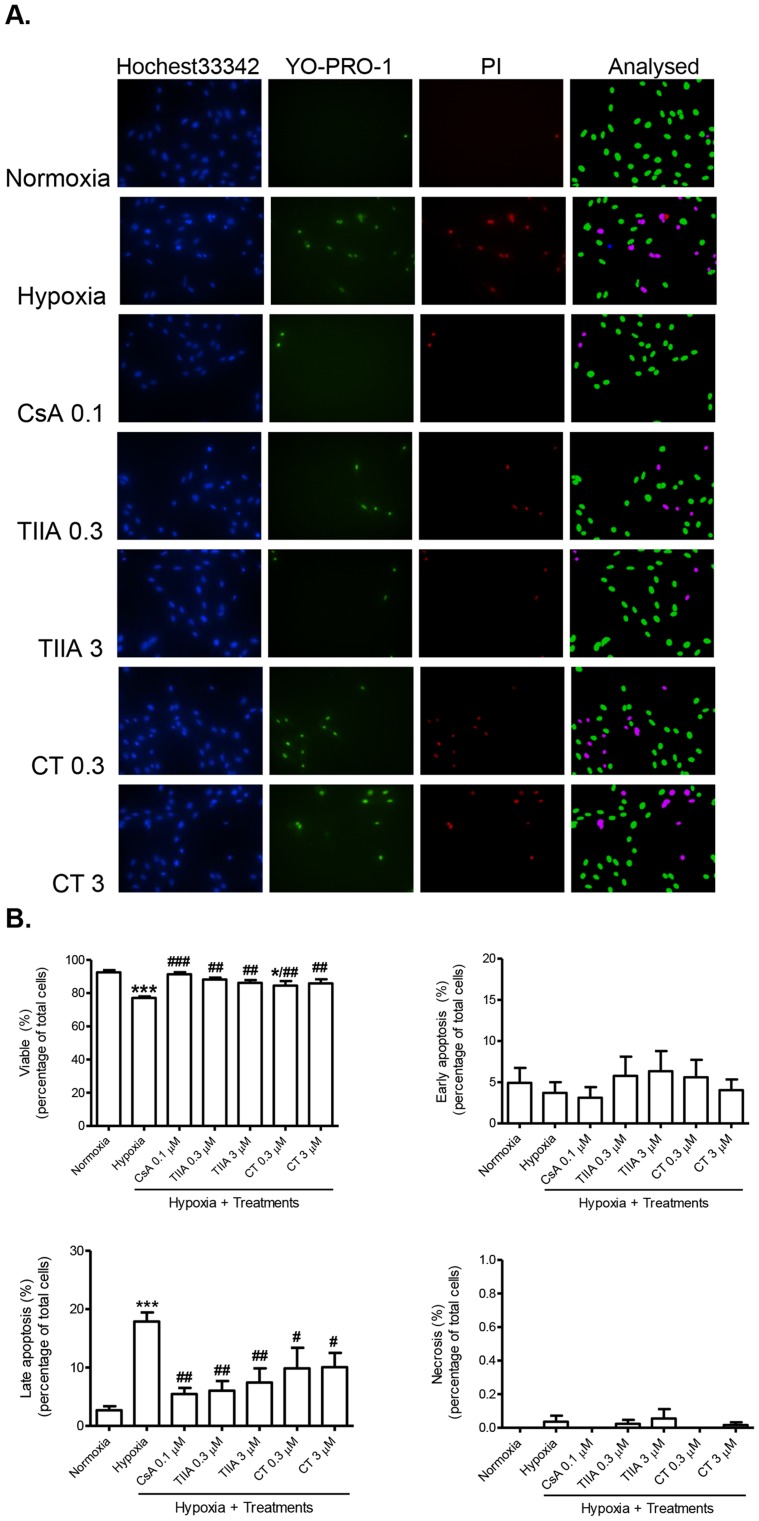
Effects of TIIA, CT and cyclosporin A (CsA) on hypoxia induced apoptosis. (A) Representative cell images from H9c2 cells staining with Hoechst 33342 (blue), YO-PRO-1 (green) and PI (red) as described in method. Analysed images show viable (green), early apoptosis (blue), late apoptosis (pink) and necrosis (red) cells respectively. (B) Mean data show subpopulations of viable cells (Hoechst 33342+), early apoptotic cells (Hoechst 33342+ and YO-PRO-1+), late apoptotic cells (Hoechst 33342+, YO-PRO-1+, PI+) and necrotic cells (Hoechst 33342+ and PI+) respectively. Values shown are mean ± SEM from n = 5. * P<0.05 vs. normoxia, # P<0.05 vs. hypoxia, ## P<0.01 vs. hypoxia, *** P<0.001 vs. normoxia, ### P<0.001 vs. hypoxia.

**Figure 4:**
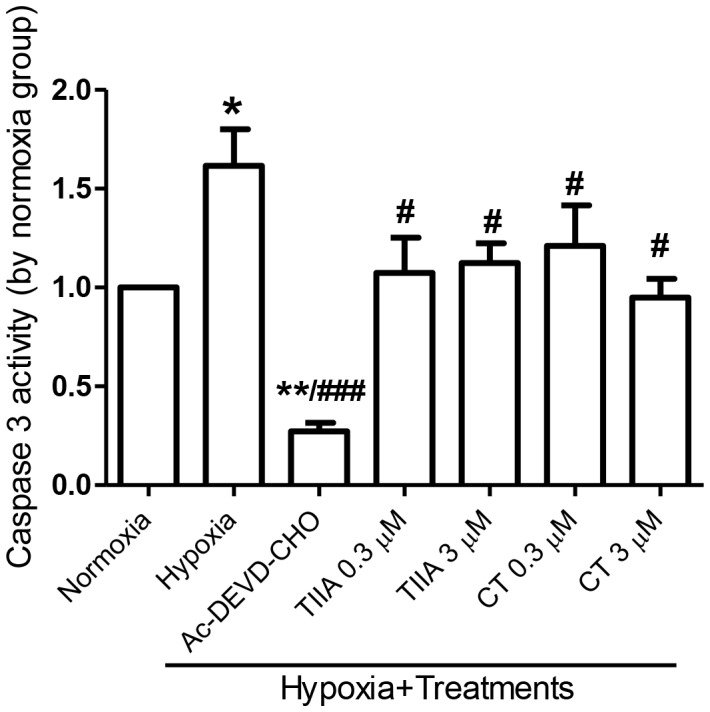
Effects of TIIA, CT and a caspase 3 inhibitor Ac-DEVD-CHO (20 μΜ) on hypoxia induced caspase 3 activation. The values of caspase 3 activity were normalized by normoxia group. Values shown are mean ± SEM from n = 4, * P<0.05 vs. normoxia, # P<0.05 vs. hypoxia, ** P<0.01 vs. normoxia, ### P<0.001 vs. hypoxia.

To examine the chronic hypoxia induced cellular morphological changes and the types of cell apoptosis, we assessed the cell apoptosis/necrosis by a fluorescence assay. Three fluorescence probes (Hoechst 33342, YO-PRO-1 and propidium iodide) were used to distinguish the cell damages by the extent of nuclear chromatin condensation and plasma membrane permeability. Analysed images of viable (green), early apoptotic (blue), late apoptotic (pink), and necrotic cells (red) are shown in Figure 3A. In normoxia cells, a high cell viable rate (92.5±3.1%) was observed, with 4.9±4.1% of early apoptosis, 2.7±1.6% of late apoptosis, and no necrosis. In contrast, hypoxia cells showed a significant increase in late apoptosis (17.9±3.5%), although the changes of early apoptosis and necrosis rate were not significant (Figure 2B). Hypoxia cells pre-treated with TIIA and CT (0.3 and 3 µM) showed a significant increase in the cell survival rate and attenuated late apoptosis rate, an effect similar to cyclosporin A (CsA, 0.1 µM), an inhibitor of MPTP opening (Figure 3B). In addition, the caspase 3 activity, a key stimulator of cell apoptosis in intrinsic pathway, was significantly activated (by 1.6 fold) under the hypoxia condition. The hypoxia-induced caspase 3 activation was significantly inhibited by both TIIA and CT (0.3 and 3 µM), as well as specific caspase 3 inhibitor, Ac-DEVD-CHO (20 µM) (Figure 4).

### Effects of TIIA and CT on Hypoxia Induced of Mitochondrial Membrane Potential

**Figure 5:**
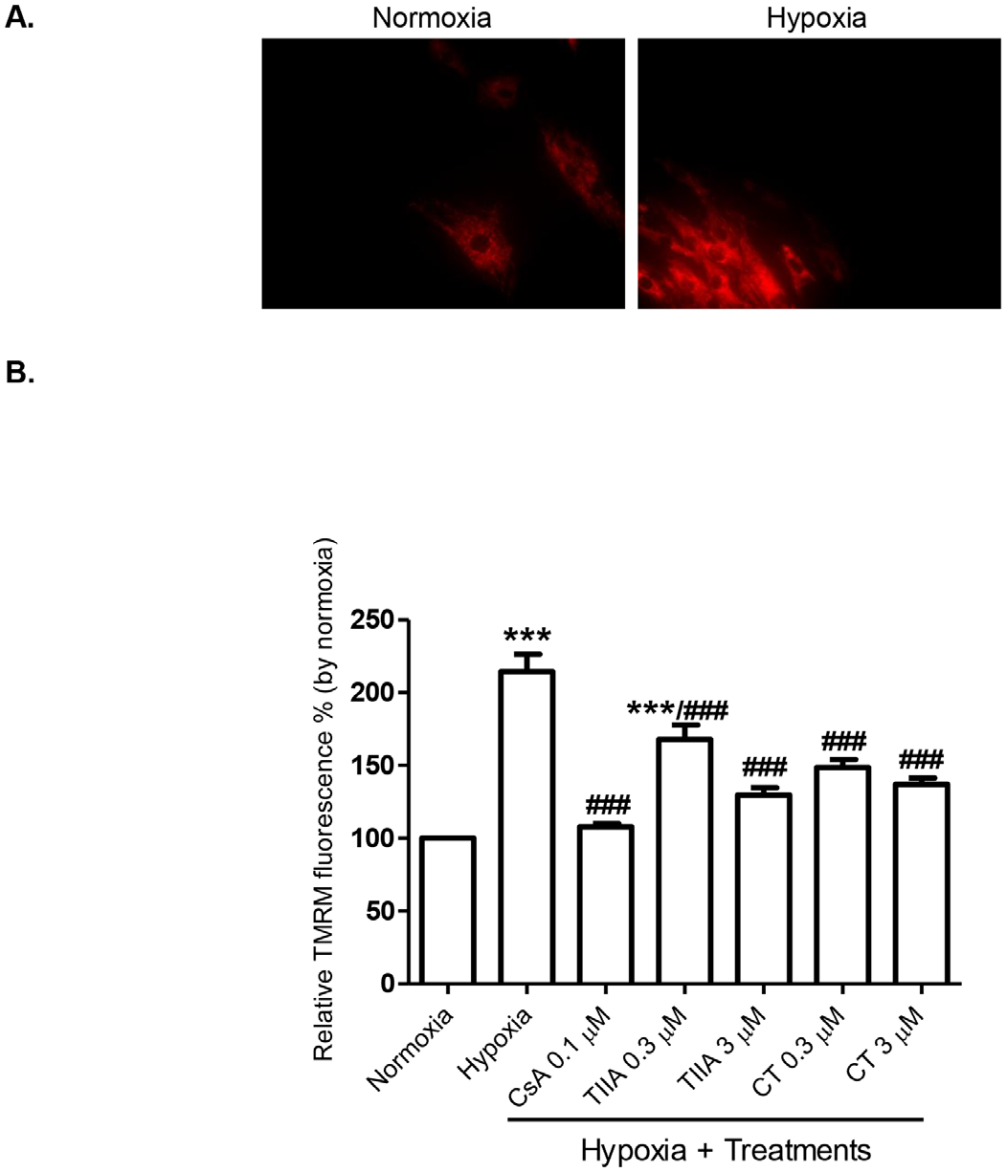
Effects of TIIA, CT, and CsA on hypoxia induced hyperpolarization in mitochondrial membrane. (A) Images of cells stained with TMRM in normoxia control cells and cells after 8 hr hypoxia. (B) Quantitative data of TMRM fluorescence intensity measured by microplate reader. Values shown are mean ± SEM from n = 3, *** P<0.001 vs. normoxia, ### P<0.001 vs. hypoxia.

To determine the association of cell apoptosis with MPTP, we measured the change of mitochondrial membrane potential as an indicator of MPTP opening. H9c2 cells were loaded with a mitochondria selective, cell-permeant and cationic fluorescent probe, TMRM. As shown figure 5A and B, cells exposed to 8 hr hypoxia exhibited a significant increase in membrane potential (2.1 fold increase) compared to normoxia group, indicating a hyperpolarization of mitochondria membrane. Hypoxia induced hyperpolarization was significantly less when the cells pre-treated with TIIA and CT (0.3 and 3 µM) (Figure 5B). For example, 3 µM TIIA group (129.6±8.9%) preserved membrane potential similar to the normoxia group. The mitochondria hyperpolarization was also inhibited by CsA (0.1 µM), an inhibitor of MPTP.

### Effects of TIIA and CT on Hypoxia Induced Cytochrome C Translocation

**Figure 6:**
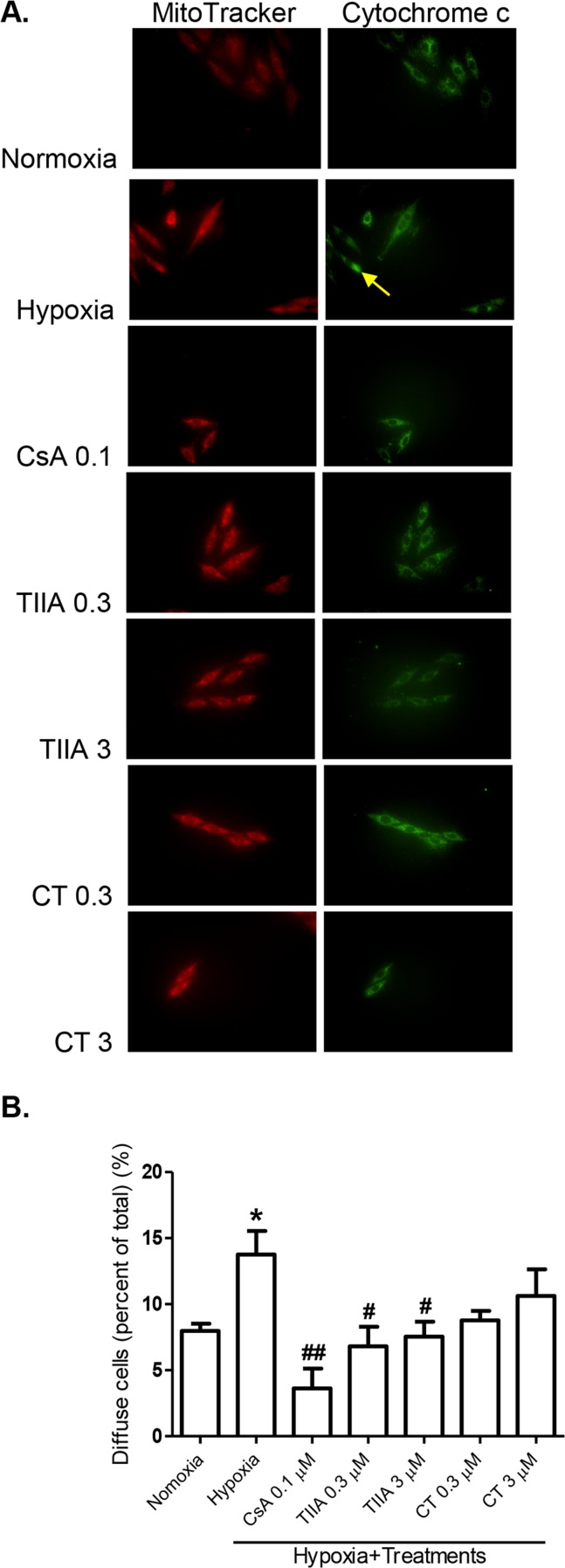
Effects of TIIA, CT and CsA on hypoxia induced cytochrome c translocation. (A) Cell images illustrate mitochondria labelling with MitoTracker Orange CMTMRos (MitoTracker) (red) and cytochrome c (green) in H9c2 cells under normoxia and hypoxia conditions, and the effects of TIIA, CT and CsA treatments (yellow arrow: cytochrome c release). (B) Quantitative data of cytochrome c diffusion rate. H9c2 cells were scored as having cytochrome c in mitochondria defined by punctuate (no cytochrome c release) or cytochrome c release to cytosolic distinguished by diffused staining and overtaking mitochondrial staining. Mean data from 500 cells per group in 3 independent experiments. * P<0.05 vs. normoxia, # P<0.05 vs. hypoxia, ## P<0.01 vs. hypoxia.

Cytochrome c release from mitochondria by opening of MPTP is a critical event promoting intrinsic death pathway by apoptotic protease activating factor 1 (Apaf-1) mediated caspase 3 activation and apoptosis [20]. We next examined the effects of TIIA and CT on cytochrome c translocation during the hypoxia. The cytochrome c translocation was determined by immunofluorescence assay as described in method. In the normoxia cells, cytochrome c displayed punctuates cytoplasmic staining, which is in agreement with its localization in mitochondria. Cells underwent hypoxia illustrated a diffused cytoplasmic staining as results of mitochondrial integrity loss (Figure 6A). In the hypoxia cells, there was a significant increase in cytochrome c translocation (1.72 fold of the normoxia control), which was significantly inhibited by TIIA (0.3 and 3 µM) and CsA (0.1 µM) (Figure 6B). CT also showed a tendency to inhibit the cytochrome c translocation, although it did not reach the statistically significant level.

### Effects of TIIA and CT on Bcl-2 Proteins Expression

**Figure 7:**
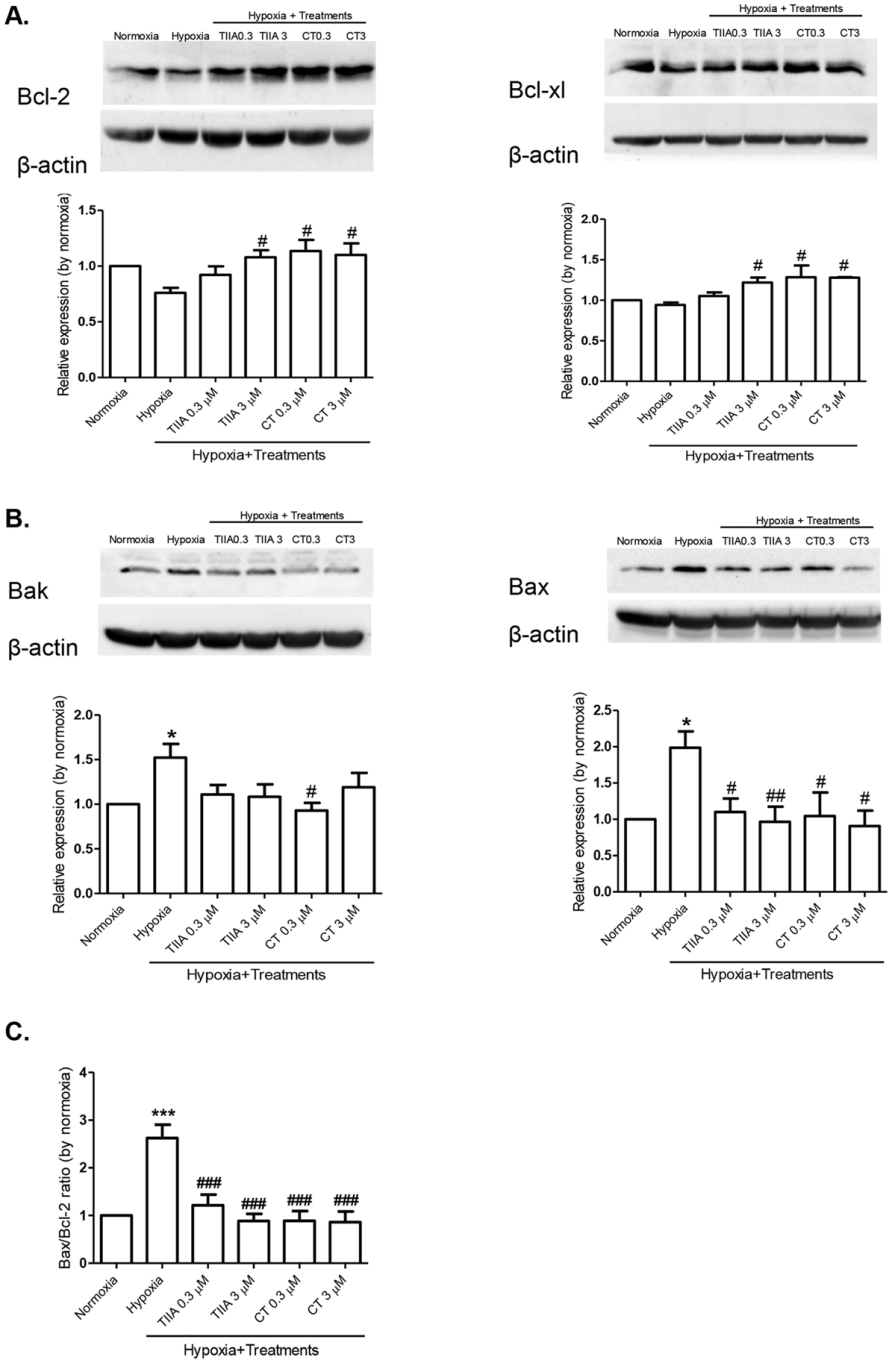
Effects of TIIA and CT on expressions of Bcl-2 family proteins on hypoxia induced H9c2 cells. (A) Anti-apoptotic proteins expression: Bcl-2 protein (50 µg) and Bcl-xl protein (20 µg) expression. (B) Pro-apoptotic proteins expression: Bak and Bax proteins expression (80 µg). (C) Bax/Bcl-2 ratio. Data shown are representative mean ± SEM of 4–5 independent experiments. * P<0.05 vs. normoxia, # P<0.05 vs. hypoxia, ## P<0.01 vs. hypoxia, *** P<0.001 vs. normoxia, ### P<0.001 vs. hypoxia.

The balance of anti- and pro-apoptotic proteins in Bcl-2 family plays an important role in the control MPTP opening and cell survival against ischemia reperfusion injuries [20]. To determine whether hypoxia induced cell apoptosis is associated with Bcl-2 family proteins, the expression of anti-apoptotic (Bcl-2 and Bcl-xl) and pro-apoptotic (Bax and Bak) proteins were determined by western blot. There was a tendency of decrease in expressions of Bcl-2 and Bcl-xl protein expressions under the hypoxia condition, although the changes were not reached the statistically significant level (Figure 7A). In contrast, expressions of Bax and Bak were significantly increased after 8 hr hypoxia (Figure 7B). Cells treated with TIIA (3 µM) and CT (0.3 and 3 µM) showed a significant increase in the expressions of Bcl-2 and Bcl-xl, but a decrease in expressions of Bax and Bak (Figure 7A and B), compared to the hypoxia control, thus the increase in Bax/Bcl-2 ratio induced by hypoxia was inhibited by both TIIA and CT (Figure 7C).

## Discussion

The main finding of the present study is that TIIA and CT protected against apoptosis in H9c2 cells induced by chronic hypoxia via acting on the mitochondrial apoptosis signalling pathway. To our knowledge, this is the first evidence on the molecular mechanism of tanshinones on protecting chronic hypoxia induced apoptosis in H9c2 cells.

Myocardial hypoxia is a main cause of cardiac dysfunction due to its triggering cell injury, apoptosis and/or necrosis [1], [21]. Under the present experimental condition, exposure of H9c2 cells to an 8 hr hypoxia caused a significant cell injury and changes in cell signalling, including cell apoptosis rate, HIF-1α activation, disturbance of mitochondria membrane potential, and caspase 3 activation, cytochrome c translocation, and imbalance of anti- and pro-apoptotic proteins in Bcl-2 family proteins, indicating an activation of mitochondria apoptosis pathway. The present observation is consistent with previous studies on apoptosis signalling pathways in H9c2 cells under hypoxia conditions [22], [23]. It is likely that the main cause of cell injury or death under the chronic hypoxia condition was due to late apoptosis, as characterised by condensed chromatin and nuclear, and changes in permeability of cell membrane which can cause mitochondrial membrane disturbance. This is supported by other findings on cytochrome c translocation and caspase 3 activation in doxorubicin induced cell apoptosis [24] and I/R injuries in rats [25].

The observed cell apoptosis and changes in signalling molecules are most likely to be associated with mitochondrial dysfunction, as demonstrated with a change in mitochondria membrane potential as an indication of MPTP opening, as reported previously [26]. The disturbance in mitochondria membrane potential may be resulted from altered mitochondria transport of proton in electron transport chain and manifested as de- and hyper-polarization [27]. The finding that TIIA and CT treatments prevented or reduced the hypoxia induced mitochondria membrane hyperpolarization may be related to their effects on mitochondria apoptosis signalling pathway (e.g. via cytochrome c release and caspase 3 activation and Bax/Bcl-2 proteins). The findings provide an explanation of cardioprotective actions of tanshinones against cell injuries involving mitochondria pathway. Previous studies have reported anti-apoptotic actions of TIIA against I/R injuries and angiotensin II induced apoptosis in PC12 cells [28] and neonatal cardiomyocytes [29] respectively, by suppressing caspase 3 activity and increasing Bcl-2 expression. TIIA has also been shown to regulate mitochondrial membrane potential in β-amyloid induced cortical neurons apoptosis [30]. Similarly, the anti-apoptotic effects of CT has been shown previously with conserving mitochondrial membrane potential, reducing Bax/Bcl-2 ratio and inhibiting cytochrome c release in nitric oxide induced neuroblastoma cells apoptosis [31]. It is possible that the anti-apoptotic actions of TIIA and CT observed in the present study are associated with not only direct action of these compounds on the intrinsic pathway and but also the antioxidant actions of tanshinones [15], [24], as there are evidence that the hyperpolarization of mitochondria membrane regulates mitochondrial ROS generation [32], [33]. Further studies are necessary to elucidate the role of ROS in the protection by tanshinones of chronic hypoxia-induced cell injury in H9c2 cells.

It has been well established that Bcl-2 family proteins play an important role in maintaining mitochondrial function and mediating cardiac protections of various therapeutic agents [34]. Overexpression of Bcl-2 has been linked to protection of cell apoptosis by inhibition of cytochrome c releases [35], while depletion of Bax protein has been shown to reduce cell death in ischemia reperfusion injury in mice [36]. TIIA has been demonstrated to decrease Bax/Bcl-2 ratio in a rat model of ischemia reperfusion injuries *in vivo* [25], and to regulate Bax/Bcl-2 ratio in neonatal cardiomyocytes treated with doxorubicin [15]. In the present study, we demonstrated that both TIIA (3 µM) and CT (0.3 and 3 µM) significantly inhibited Bax and Bak expressions, but increased Bcl-2 and Bcl-xl expressions under the chronic hypoxia condition, resulting in a reduced Bax/Bcl-2 ratio and increased cell survival rate. Interestingly, under the hypoxia condition, the Bak expression was markedly elevated, while that of Bcl-xl was not significantly changed. Previous studies showed that Bak protein could be sequestered not only by Bcl-xl but also by Mcl-l (a member of anti-apoptotic proteins of Bcl-2 family proteins) [37]. Thus, it is possible that the Bak protein activation in the present study may involve inactivation of Mcl-1 protein by chronic hypoxia. It is not clear if the change of Bak expressions by TIIA and CT in this study was resulted from their actions on Bcl-xl protein or involved Mcl-1 protein. Further study is necessary to confirm the further mechanism involved.

Previous studies have reported that increased HIF-1α and HIF-regulated gene expressions by hypoxia are associated with cell apoptosis, possibly by stimulation of Bnip3 protein and p53 protein which subsequently influences in Bax and Bcl-xl proteins in mitochondria [38]. The elevated Bax and HIF-1α expressions as observed in the present study indicate a possible link between these signalling molecules. This is also supported by our observation in a separate microarray study that showed significant increase in Bnip3 gene expression in H9c2 cells under the same hypoxia condition (Data not shown), indicating a possibility of involving HIF-1α activation via p53 upregualtion on Bax upregulation induced by hypoxia. Interestingly, TIIA has been shown to decrease HIF-1α protein translocation induced by lipopolysaccharide (LPS) in mice lung [39] and down regulating p53 protein expression in PC12 cells [40]. Thus it is possible that TIIA may act via HIF-1α dependent p53 signalling pathway in H9c2 cells.

In conclusion, we have demonstrated that TIIA and CT can prevent chronic hypoxia induced H9c2 cells apoptosis by targeting mitochondrial death pathway via balancing in anti- and pro-apoptotic proteins in Bcl-2 family proteins and inhibition of mitochondria membrane hyperpolarization, cytochrome c translocation, caspase 3 activity and inactivation of HIF-1α protein. These mechanisms may be involved in mediating cardiac protective actions of tanshinones in hypoxia and ischemic conditions.

## Materials and Methods

### Chemicals

Tansinone IIA (TIIA) and cryptotanshinone (CT) were purchased from National Institute for the Control of Pharmaceutical and Biological Products (>99% purity) (Beijing, China). Dulbecco’s Modified Eagle’s Medium (DMEM), fetal bovine serum (FBS), penicillin and streptomycin were purchased from Gibco BRL (NY, USA). GasPak™ EZ Anaerobe Container System Sachets with Indicator and GasPak™ EZ Standard Incubation Container were from Becton Dickinson and company (Sydney, Australia). Trypsin-EDTA solution, Bradford reagent, Bicinchoninic Acid Kit, protease inhibitor cocktail, Hoechst33342, propidium iodide (PI), cyclosporin A (CsA), dimethyl sulfoxide, tetramethylrhodamine methyl esters (TMRM), 3-(4,5-dimethylthiazol-2-yl)-2,5-diphenyltetrazolium bromide and caspase 3 assay kit (colorimetric) were from Sigma-Aldrich (MO, USA). HIF-1α, Bcl-2, Bcl-xl, cytochrome c and β-actin antibodies, goat anti-mouse IgG-HRP and goat anti-rabbit IgG-HRP were product of Santa Cruz Biotechnology. Bax and Bak were purchased from Cell signaling Technology (MA, USA). Alexa Fluor ®488 conjugated goat anti-mouse IgG, YO-PRO-1, DAPI and MitoTracker Orange CMTMRos was purchased from Molecular Probes (San Francisco, USA). SuperSignal West Pico Chemiluminescent substrate and NE-PER Nuclear and Cytoplasmic Protein Extraction Reagents were purchased from Thermo Scientific (Rockford, USA).

### Cell Culture and Hypoxia

The H9c2 embryonic rat heart-derived cells were obtained from American Type Culture Collection (ATCC; VA, USA) and maintained in Dulbecco’s modified Eagle’s medium supplemented with 10% v/v fetal bovine serum and 100 *µ*g/ml penicillin/streptomycin at 37°C in a humidified atmosphere containing 5% CO_2_ (passage 25–35).

To induce hypoxia, cells were placed in a GasPak EZ Gas generating Pouch System for 8 hr and incubated with serum free and glucose free DMEM as described previously [41]. As normoxia control, serum free DMEM was added to cells and incubated for 8 hr in normoxia condition (21% O_2_).

### Drug Treatment

Cells were treated with TIIA or CT (0.3 and 3 µM), respectively, 2 hr before inducing the hypoxia and during the hypoxia period. The experimental condition was established from a preliminary study involving different concentration of tanshinones (0.1–10 µM) at different (2 and 24 hr) pre- and post-hypoxia incubation periods. In some experiments, CsA (0.1 µM) was added 1 hr before induction of hypoxia as a positive control. Ac-DEVD-CHO (20 μΜ), caspase 3 inhibitor, was treated 30 mins before adding the caspase 3 substrate.

### MTT Assay

To determine effects of TIIA and CT on cell viability in H9c2 cells, MTT (3-(4,5-dimethylthiazol-2-yl)-2,5-diphenyltetrazolium bromide) assay was performed as described previously [42]. Briefly, cells (5×10^4^ cells/ml) were seed in 96-wells. After 2 days of culture, TIIA and CT (0.01 and 10 µM) were added and incubated for 24 hr. At the end of the incubation, MTT solution was added to each well at a final concentration of 0.5 mg/ml and incubated for 4 hr at 37°C. Then, the culture medium was discarded and 150 µl DMSO was added to each well to dissolve dark blue formazan crystals. The absorbance was read at 570 nm using FLUOstar Omega plate reader (BMG LABTECH, Ortenberg, Germany).

### Apoptosis and Necrosis Assay

Cell apoptosis and necrosis were assessed by using a combined fluorescent staining with Hoechst 33342, YO-PRO-1and propidium iodide (PI) as previously described [43]. Briefly, at the end of hypoxia period, Hoechst 33342 (0.5 µg/ml), YO-PRO-1 (0.1 µM), and PI (0.1 µg/ml) were added to each well and incubated for 30 mins at 37°C. The cell images were automatically obtained using Image Xpress MICRO system (Molecular Devices, CA, USA) at 20X magnification with binning of 1 and gain of 2 using laser-based focusing. Images were captured using a DAPI filter (350/70 nm Ex, 470/50 nm Em for Hoechst 33342), GFP filter (490/40 nm Ex, 510/50 nm Em for YO-PRO-1) and Cy3 filter (550/35 nm Ex, 570/30 nm Em for PI). The cell images were analysed by using Cell Health program in Meta express software (Molecular Devices).

### Caspase-3 Activity Assay

Caspase-3 activity was determined by colorimetric assay kit (Sigma Aldrich, MO, USA) according to manufacturer’s instruction. Briefly, after the hypoxia period, cells were incubated with the lysis buffer (1×10^7^/100 µl) for 30 mins and the lysate were centrifuged at 10,000 g for 5 mins. Protein concentrations in supernatant were determined by Bradford protein assay. Fifty µl of cell lysate containing 100 µg/50 µl protein were incubated with 50 µl assay buffer including 200 µM caspase 3 substrate *(N*-acetyl-Asp-Glu-Val-Asp-*p*-nitroaniline(Ac-DEVD-pNA)) for 4 hr at 37°C. The amount of free pNA (yellow colour) was determined by FLUOstar Omega microplate reader (BMG LABTECH) at 405 nm, which is proportional to the amount of DEVDase (caspase-3) activity present in the sample.

### Western Blot Analysis

For whole cell lysate preparations, cultured H9c2 cells were harvested by scraping and centrifugation, washed with PBS, and re-suspended in 100 µl/10^6^ cells RIPA buffer (150 mM NaCl, 1% Nonider P-40, 0.5% deoxycholate, 0.1% sodium dodecylsulfate, 50 mM Tris-hydrochloric acid, 2 mM phenylmethylsulfonylfluoride, and protease inhibitor cocktail, pH 7.4). The cell lysate was homogenised by pipetting and incubated on ice for 20 mins. After the incubation, cell lysate was centrifuged at 13,000 g for 10 mins. Supernatant was collected and stored at −80°C till use. The protein content was measured by bicinchoninic acid protein assay. To specify HIF-1α translocation and stabilization, nuclear fractions were subjected to western blot. Nuclear fractions were isolated according to manufacture’s instruction using NE-PER Nuclear and Cytoplasmic Protein Extraction Reagents (Thermo Scientific, Rockford, USA). The protein content was measured by Bradford protein assay.

Whole cell lysates (20–80 µg) were subjected to 12% SDS-PAGE (Bcl-2, Bcl-xl, Bax and Bak) and transferred onto a nitrocellulose membrane. Nuclear fractions (25 µg) were subjected to 8% SDS-PAGE (HIF-1α) and transferred onto a nitrocellulose membrane.

After blocking with 5% nonfat milk for 1 hr at room temperature, blots were probed with HIF-1α (1∶500), Bcl-2 and Bcl-xl antibody (1∶500), Bax and Bak antibody (1∶1,000) at 4°C over night. Next day, the blots were incubated with second antibodies and the incubated with supersignal west pico chemiluminescent substrate (Thermo Scientific, Rockford, USA). The membrane was then placed in a Chemidoc XRS unit (BioRad Laboratories, CA, USA) and the chemiluminescence was detected by a CCD camera connected to the Chemidoc XRS unit and a digital output was obtained. The digital image was analysed for densitometry using Image Lab™ Software, Version 3.0 (Bio Rad Laboratories).

### Measurement of Mitochondria Membrane Potential

Mitochondrial membrane potential was determined using tetramethyl rhodamine methyl ester (TMRM), a lipophilic cation that selectively accumulates within mitochondria in a membrane potential depending manner [44]. Briefly, cells were incubated with 150 nM TMRM after hypoxia for 15 mins at 37°C. Then, the cells were washed 3 times with PBS gently. After the last wash, 100 µl of PBS was left in plates. Then, TMRM fluorescence was read immediately on FLUOstar Omega microplate reader with an excitation wavelength of 544 nm and an emission wavelength of 590 nm. The fluorescence intensity was normalized to cell number. To visualise TMRM accumulations, cells were incubated with 150 nM TMRM for 30 mins at 37°C after hypoxia, and then washed with media. Cells were visualised in Image Xpress MICRO system (Molecular Devices).

### Cytochrome C Translocation

Cytochrome c translocation was determined as previously described [45]. Briefly, cells were incubated with 200 nM MitoTracker Orange CMTMRos after inducing hypoxia for 20 mins at 37°C to localize the mitochondria. Then, cells were fixed in 4% paraformaldehyde for 10 min followed by permeabilization in 1% Triton X-100. After washing with PBS, cells were blocked in 20% goat serum for 30 mins at 37°C and then incubated with cytochrome c antibody (1∶100) for 1 hr at 37°C. Subsequently, cells were incubated with Alexa Fluor ®488 conjugated goat anti-mouse IgG (1∶1000) for 1 hr at 37°C. After the incubation, cells were washed 4 times with PBS and DAPI (1∶2000) was included in the second wash for 10 mins as nuclear counterstaining. The cell images were obtained using Image Xpress MICRO system (Molecular Devices) at 20X magnification with binning of 1 and gain of 2 using laser-based focusing. Images were captured using a DAPI filter (350/70 nm Ex, 470/50 nm Em for DAPI), GFP filter (490/40 nm Ex, 510/50 nm Em for Cytochrome c) and Cy3 filter (550/35 nm Ex, 570/30 nm Em for MitoTracker Orange CMTMRos).

### Statistical Analysis

Results were expressed as means ± SEM. Statistical differences among groups were analysed by one-way analysis of variance (ANOVA), and p value less than 0.05 was considered as statistically significant.
